# FunPat: function-based pattern analysis on RNA-seq time series data

**DOI:** 10.1186/1471-2164-16-S6-S2

**Published:** 2015-06-01

**Authors:** Tiziana Sanavia, Francesca Finotello, Barbara Di Camillo

**Affiliations:** 1 Department of Information Engineering, University of Padova, via G. Gradenigo 6A, 35131 Padova, Italy

## Abstract

**Background:**

Dynamic expression data, nowadays obtained using high-throughput RNA sequencing, are essential to monitor transient gene expression changes and to study the dynamics of their transcriptional activity in the cell or response to stimuli. Several methods for data selection, clustering and functional analysis are available; however, these steps are usually performed independently, without exploiting and integrating the information derived from each step of the analysis.

**Methods:**

Here we present *FunPat*, an R package for time series RNA sequencing data that integrates gene selection, clustering and functional annotation into a single framework. *FunPat *exploits functional annotations by performing for each functional term, e.g. a Gene Ontology term, an integrated selection-clustering analysis to select differentially expressed genes that share, besides annotation, a common dynamic expression profile.

**Results:**

*FunPat *performance was assessed on both simulated and real data. With respect to a stand-alone selection step, the integration of the clustering step is able to improve the recall without altering the false discovery rate. *FunPat *also shows high precision and recall in detecting the correct temporal expression patterns; in particular, the recall is significantly higher than hierarchical, k-means and a model-based clustering approach specifically designed for RNA sequencing data. Moreover, when biological replicates are missing, *FunPat *is able to provide reproducible lists of significant genes. The application to real time series expression data shows the ability of *FunPat *to select differentially expressed genes with high reproducibility, indirectly confirming high precision and recall in gene selection. Moreover, the expression patterns obtained as output allow an easy interpretation of the results.

**Conclusions:**

A novel analysis pipeline was developed to search the main temporal patterns in classes of genes similarly annotated, improving the sensitivity of gene selection by integrating the statistical evidence of differential expression with the information on temporal profiles and the functional annotations. Significant genes are associated to both the most informative functional terms, avoiding redundancy of information, and the most representative temporal patterns, thus improving the readability of the results. *FunPat *package is provided in R/Bioconductor at link: http://sysbiobig.dei.unipd.it/?q=node/79.

## Background

Understanding biological systems regulation is one of the main challenges of systems biology. In particular, gene expression regulation is an intrinsically dynamic phenomenon, whose characteristics can be investigated using dynamic expression data. In this context, time series high-throughput data provide a powerful approach to identify characteristic temporal profiles of specific biological processes and to understand the transcriptional response to stimuli. In the last years, as the sequencing costs decrease, techniques for measuring transcriptome have rapidly changed from microarray to RNA sequencing (RNA-seq). RNA-seq allows both determining transcript sequences and quantifying their abundance at the same time; thus, compared to the microarray technique, RNA-seq avoids the design of specific probes, enabling a higher number of transcripts to be measured on a wider dynamic range.

There are several issues to be considered when RNA-seq is used with a time series experimental design [1]. Current time series datasets have few biological replicates available, in general no more than three replicates [2]. Even if sequencing data avoid several noise issues related to microarray data, as background and cross-hybridization noise, they still need an estimate of the biological variability within the groups, otherwise there is no statistical basis for inference of differences across time and between different experimental conditions [3]. Once the transcript counts are generated, another important issue to be considered is data normalization, which is particularly critical for RNA-seq time series data since gene expression has to be monitored on the same scale in every time point in order to correctly identify temporal patterns of gene expression. In fact, given a transcript having the same expression level in two different samples, the probability that a read measured in a sample comes from that specific transcript depends on both the relative abundance of the transcript with respect to all the other transcripts and the total number of reads in the sample [4,5]. To remove these biases, several normalization methods are considered in the literature. Among others, Trimmed Mean of M-values (TMM) [6] provides scaling factors to correct the library sizes calculated as a weighted mean of log ratios after filtering out the most expressed genes and the genes with the largest log ratios. This approach has been recently shown to prevent loss of statistical power in the analysis of RNA-seq data when high-count genes are present [7].

Once data are pre-processed, the typical workflow to analyze time series expression data includes: i) the selection of the differentially expressed (DE) genes; ii) a clustering step to summarize the information using a limited number of representative profiles; iii) the functional analysis to associate each cluster of genes to meaningful biological annotations.

In the context of DE gene selection, most of the approaches available for RNA-seq data, such as edgeR [8], DESeq [9], baySeq [10] and the recent distribution-free method proposed by Li and Tibshirani [11], are focused on the comparison among different groups of samples and do not take into account the inherent dependencies among samples that are characteristic of time series data. EdgeR [8] and DESeq [9], have recently enabled multifactor comparison performed using a generalized linear model, but, as pointed out in a recent review [2], this kind of approach is independent from the order of the time points, thus ignoring the overall dynamics. Moreover, a general issue, common to both static and dynamic high-throughput expression studies, is the control of the type I error rate. In order to control this error rate in a multiple-testing fashion, e.g. using False Discovery Rate (FDR), stringent thresholds need to be applied, thus leading to a high number of false negatives with a consequent loss of information. This problem has become even more evident with RNA-seq data, due to the higher number of monitored transcripts with respect to the previous technologies.

As regards the clustering step, classical algorithms such as k-means and hierarchical clustering are usually applied also to RNA-seq data. These methods, however, do not account for technical and biological noise and require to set, either *a priori *or *a posteriori*, the number of clusters, the distance metric or the linkage method. Alternatively, model-based methods such as Bayesian clustering are able to overcome these drawbacks, but require a probabilistic model of data generation and are usually computationally demanding. Recently, a model-based clustering method specifically designed for RNA-seq data was proposed by Si et al. [12]. Specifically, this method assumes that data are generated by a mixture of probability distributions, either Poisson or Negative Binomial, and defines a likelihood function of the mixture models representing each gene.

Functional analysis is usually performed at the end of the entire analysis workflow using annotations from genomic databases such as Gene Ontology (GO) [13], either by simply mapping the genes to known functional terms or identifying the most enriched terms, using approaches such as the recent version of Gene Set Enrichment Analysis for RNA-seq data (SeqGSEA) [14]. Keeping the functional analysis as the last step, however, may introduce a bias in itself due to both false negative genes in the selection step and wrong clustering. Moreover, the organization of functional terms in genomic databases is usually structured according to different levels of specificity of the associated biological concepts, as it happens for example for the GO terms, introducing redundancy of information in the related annotations.

In this work, we address the above issues by integrating selection, clustering and functional analysis into a single analysis framework, implemented in the R package *FunPat*. In particular, we focus on the identification of groups of DE genes characterized by both a common temporal pattern and a common biological function. Intuitively, if a gene characterized by a significant nominal p-value is excluded by the multiple tests correction but it shares the same temporal expression pattern and the same functional annotation with a set of genes selected as differentially expressed, the gene is likely to be a false negative. As a consequence, recovering it in the pool of DE genes might increase the recall without negatively affecting the precision.

*FunPat *exploits the functional annotations of genomic databases, organizing them into *Gene Sets*, e.g. GO terms, and performing an integrated selection-clustering analysis in each Gene Set. In particular, when a hierarchical structure of the functional annotations is available, as in GO database, *FunPat *searches for temporal patterns starting from the most specific Gene Sets and, whenever present, removes the genes selected as DE from the Gene Sets representing more general biological concepts. The output of *FunPat *is a list of Gene Sets, each characterized by different temporal patterns and the corresponding lists of DE genes.

To the best of our knowledge, there are few approaches in the literature that have been implemented for or applied to time series RNA-seq data. Recently, Wu and Wu [15] proposed a unified approach to model gene profiles based on Functional Principal Component Analysis (FPCA) technique. The method was originally tested on microarray data, but it was recently applied to RNA-seq data [16]. Another approach originally designed to model temporal gene expression from microarray data, maSigPro (MicroArray Significant Profiles), was recently updated to handle time series RNA-seq data and it is based on two steps of modelling based on generalized linear regression [17]. Both FPCA and maSigPro do not use any prior information from functional databases.

In the following, we present *FunPat *and assess gene selection and clustering performance on a number of simulated datasets with known DE genes and dynamic profiles, in comparison with maSigPro, FPCA, edgeR and the hierarchical, k-means and model-based clustering proposed in [12]. We also consider edgeR in the analysis because, although not explicitly designed for time series data, the generalized linear model (GLM) allows analyzing complex experimental designs such as dynamic experiments. To better appreciate the various facets of *FunPat *and compare it with the other methods also on real data, we consider two different datasets, one describing the temporal response of B cell samples from different vaccinated subjects [16] and the other one representing the pancreatic endocrine differentiation of human embryonic stem cells at defined developmental stages [18].

## Methods

### FunPat pipeline

**Figure 1:**
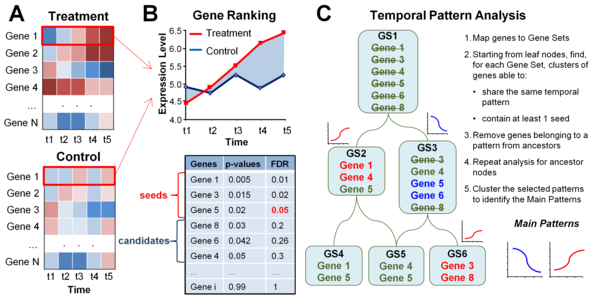
**Description of FunPat workflow**. Starting from time series expression data monitored in two different conditions (A), the Bounded-Area method provides a rank of genes according to a statistic built from the experimental replicates (B). Both *seeds *and *candidates *are mapped to structured prior knowledge organized into Gene Sets (GS) and a model-based clustering is applied to each Gene Set, following the procedure described in C. The pipeline identifies both Gene Set-specific patterns, characterizing clusters of genes (e.g. Gene 1 and 4 for GS 2), and Main Patterns, characterizing clusters of Gene Sets (e.g. red Main Pattern, associated to GS 2 and 6).

*FunPat *takes as input the expression data and the functional annotations organized according to Gene Sets, which can be defined as GO terms, pathways or other sets depending on the annotation database used as input. A summary of *FunPat *analysis pipeline is displayed in Figure 1. First, *FunPat *assigns a p-value to each gene based on the Bounded-Area method described in Di Camillo et al. [19]. For each gene, the area *A *of the region bounded by the gene transcriptional expression profiles in two experimental conditions (e.g. treatment *vs*. control), or in a single condition *vs*. a baseline, is calculated (Figure 1A, B). A p-value is assigned to *A *by evaluating its statistical significance against a null hypothesis distribution. To do that, the available replicates are exploited to model the biological-plus-technical variability and its dependency on the mean gene expression level. This model is then used to build the null hypothesis distribution of *A*, named *A*^H0^, by a Monte Carlo resampling approach. This method requires only at least two replicates for a single time point, thus addressing lack of fully replicated time series experiments, typical of currently available RNA-seq data. Finally, different distribution models (Gamma, Log-normal, Weibull) are used to fit the entire set of *A*^H0 ^values and the best model is chosen according to the goodness of fit and the parameter precision. Quantiles of the empirical distribution can be also used as an alternative to the above models.

Exploiting the p-values assigned by the Bounded-Area method, *FunPat *performs a gene selection simultaneously to a Gene Set-specific clustering. First, two sets of genes are defined: *seeds*, i.e. genes passing a FDR threshold chosen by the user, and *candidates*, i.e. genes passing a soft-threshold applied to not-adjusted p-values (Figure 1B). Alternatively, *seeds *and *candidates *can be provided as input by the user. To identify the main transcriptional dynamics characterizing the expression data, *FunPat *searches for the common temporal patterns on groups of genes belonging to the same Gene Set (Figure 1C). The core of this analysis is a linear model-based clustering [20], which searches for a cluster of genes whose time series expression profile *X = <x(1),..., x(M)>*with M time points can be modelled by the following equation:

(1) X=k⋅P+q+Σ

where *P = <p(1),..., p(M)>*is the characteristic temporal pattern, *k *and *q *are the gene-specific parameters of the model and *Σ *is the error covariance matrix. The clustering algorithm iteratively performs an identification step of the gene-specific parameters and a temporal pattern search using an Expectation-Maximization approach. A cluster is considered significant if it contains at least one *seed *gene and passes both a goodness of fit and a runs test. Further details of the clustering algorithm are reported in Additional File 1.

At the end of the analysis, *FunPat *considers a gene as significantly differentially expressed if either it is a *seed *gene or it belongs to a significant pattern. Intuitively, since each pattern is required to contain at least one *seed *gene, all genes associated to the same pattern are likely to be differentially expressed because they are highly correlated to the same temporal profile and, since the clustering is specific for each Gene Set, they share a common biological function or pathway.

Gene Sets used in *FunPat *pipeline can be organized according to a hierarchical structure, such as the direct acyclic graph (DAG) in GO database. Structured annotations provide further useful information: relationships between the biological terms in a hierarchical structure codify for the specificity of some terms with respect to others and can help to associate genes to the most informative terms avoiding redundant annotations. When structured information is available, *FunPat *assumes that genes annotated to a Gene Set are also annotated to all its ancestors and that the farther the Gene Set is from the root node, the more specific information it conveys. *FunPat *performs the clustering starting from the Gene Sets associated to the most specific terms and then removes all the genes belonging to a significant pattern from the ancestor nodes, similarly to what has been proposed by Alexa et al. [21] in the context of the functional enrichment analysis.

Since similar temporal patterns can be identified for different Gene Sets, *FunPat *applies a second clustering step to identify the Main Patterns characterizing the data. Each Main Pattern thus represents groups of Gene Sets characterized by highly-correlated temporal patterns of DE genes (Figure 1C).

### FunPat implementation

**Figure 2:**
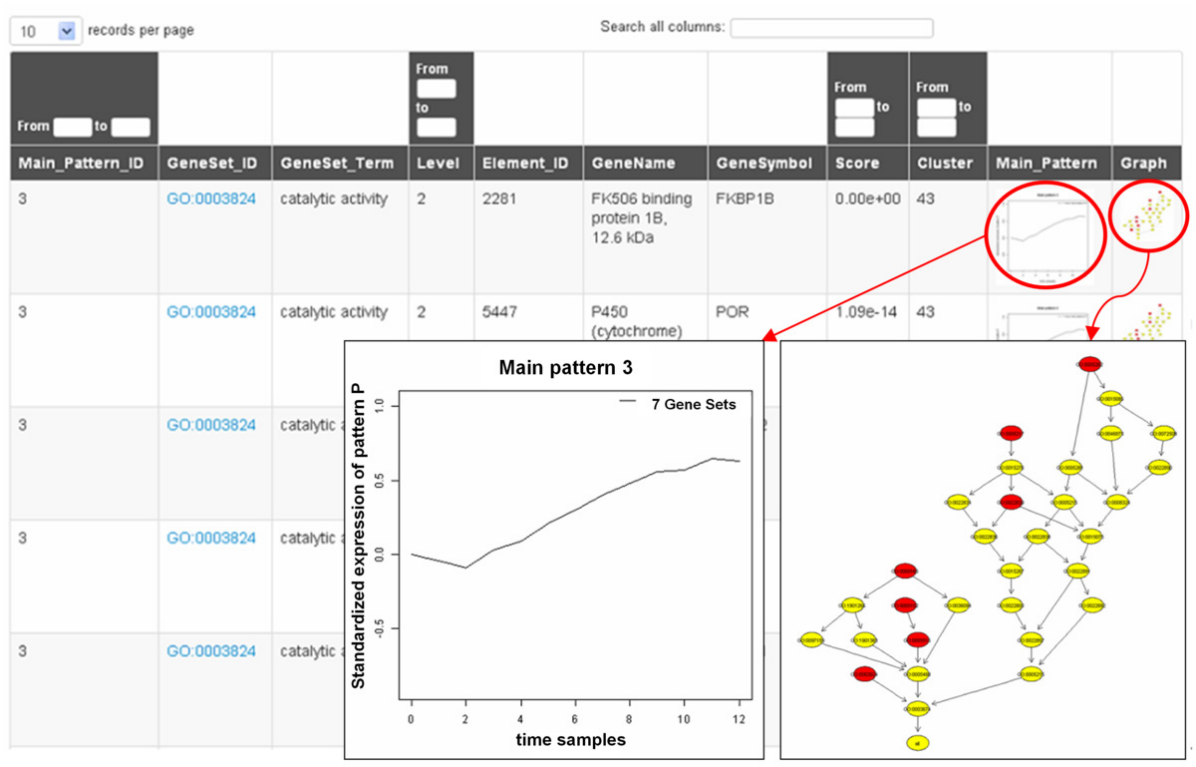
**FunPat HTML report for Main Patterns**. *FunPat *generates an HTML table reporting, for each gene, the associated Gene Set (GO term in the example), the plot of the temporal Main Pattern and the position of the terms sharing the same Main Pattern in the hierarchical structure. In the graph, red nodes indicate the selected Gene Sets associated to the Main Pattern, whereas the yellow nodes are the available nodes in the graph used to connect all the selected Gene Sets.

*FunPat *is provided in R/Bioconductor with the related documentation, it is open source under the GPL-3 License and it requires R version 3.0.3 or higher to perform the analyses. As output, *FunPat *reports the selected genes and Gene Sets into simple tab delimited .txt files and plots the temporal patterns on separate .pdf files. The package allows the user to easily display the results into HTML tables with sortable and filterable columns, plots and hyperlinks to other data sources such as NCBI and GO databases. Figure 2 shows an example of the final report summarizing the information on the identified Main Patterns. Other two HTML reports are generated by *FunPat *displaying the output of the Bounded-Area method and the temporal pattern profiles associated to each Gene Set (Additional File 2).

### RNA-seq time series data simulation

**Figure 3:**
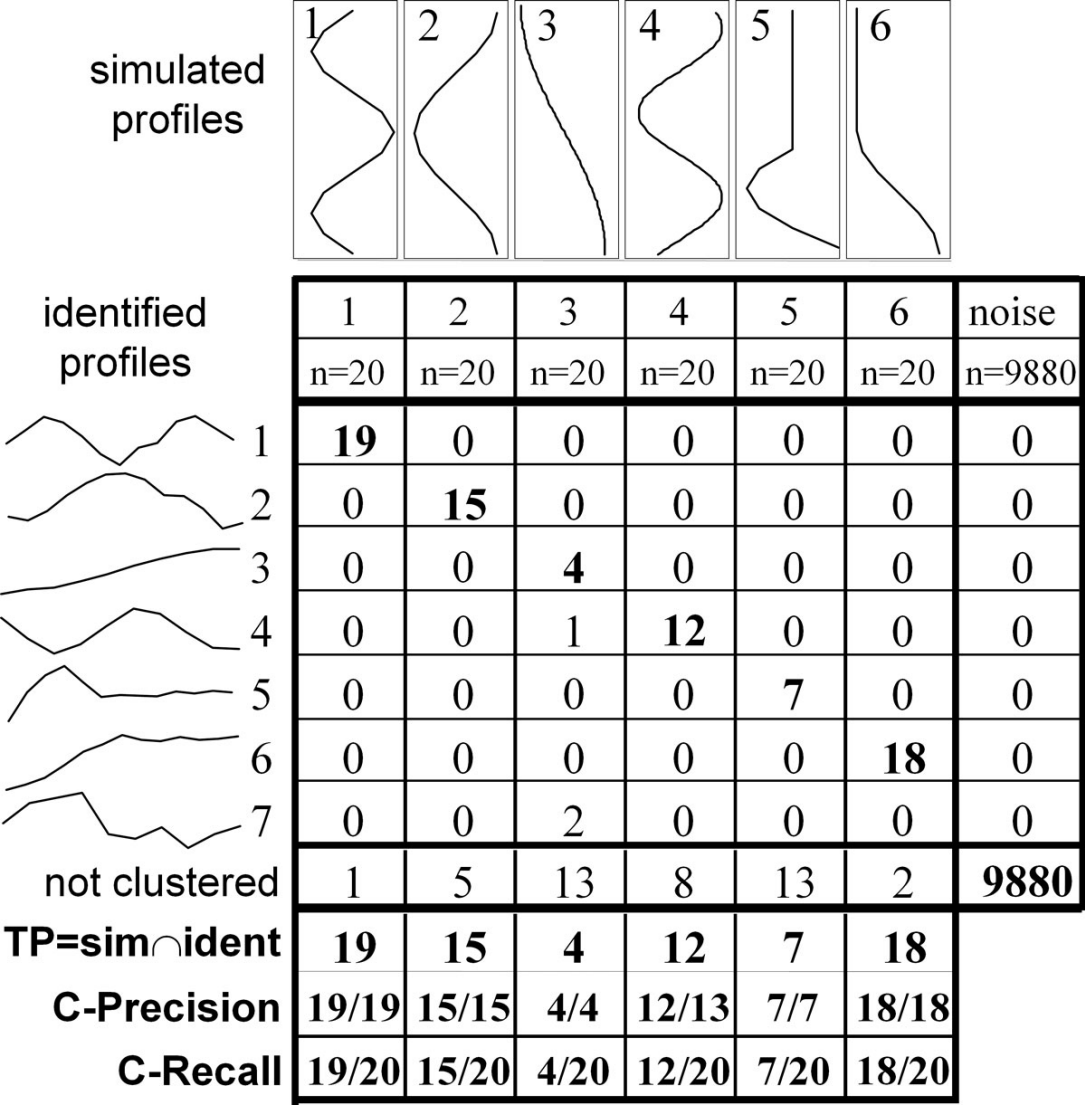
**Example of C-precision and C-recall**. Toy example illustrating how C-precision are C-recall are calculated. Each simulated dataset, like the toy example shown in this figure, consists of 120 differentially expressed genes separated in 6 clusters characterized by different temporal patterns and 9880 not differentially expressed profiles. C-precision is calculated as true positives divided by the number of genes identified in the cluster; C-recall as true positives divided by the number of genes in the simulated cluster. As an example, since all the genes belonging to the identified profile 3 also belong to the same simulated pattern 3, the C-precision associated to the identified profile is 1 (4/4). On the other hand, the pattern 3 is also associated to other 16 genes which do not belong to this profile, thus the corresponding C-recall is 0.2 (4/20).

In order to assess *FunPat *pipeline performance in terms of selection of DE genes and correct identification of the temporal patterns, its application to simulated time series data was tested. 100 time series datasets were generated, simulating a dynamical response measured in two different conditions: treatment and control. Each dataset is characterized by N = 10000 genes monitored over M = 13 time samples. 120 genes were simulated as differentially expressed, belonging to S = 6 different temporal patterns (Figure 3), representing the log fold-changes of expression levels over time induced by the treatment with respect to the control.

In particular, the expression profile *θ_f_(t) *of a given gene *f *was modelled on log scale as follows:

(2) $$\text{log}\left(\theta_{f}\left(t\right)\right)=k_{f}\cdot P_{j}\left(t\right)+q_{f}\;\;t=0,...,M-1$$

where *P_j_(t) *(j = 1,..., S) is the temporal pattern reflecting the changes in gene expression levels in response to treatment and *k_f _, q_f _*are gene-specific parameters. *q_f _*was sampled from a normal distribution N(1.5, 1.8) and *k_f _*was sampled from a uniform distribution in the interval [0.5, 2]. Each pattern *P_j_(t) *was used to simulate the expression profiles of 20 DE genes, for a total of 120 DE genes. The log fold-changes of the remaining 9880 genes were simulated as:

(3) $$\text{log}\left(\theta_{f}\left(t\right)\right)=q_{f}\;\;t=0,...,M-1$$

Assuming that the genes are single-isoform and have the same length for all the simulated transcripts, the probability that a read comes from a gene *f *can be computed, for each time point *t*, as:

(4) $$\pi_{f}\left(t\right)=\;\frac{\theta_{f}\left(t\right)}{\overset{N}{\underset{f=1}{\sum }}\theta_{f}\left(t\right)}\;\;t=0,...,M-1$$

*π_f_(t) *was used to obtain the final transcripts counts, using a Negative Binomial distribution NB(R·*π_f_(t)*, φ), where R is the sequencing depth and φ is the dispersion parameter. The sequencing depth of each sample was sampled from a uniform distribution in the interval [10^6^, 10^7^] and the dispersion parameter was set to φ = 0.1. Three replicates were generated for each time point. Simulated data were finally normalized according to the TMM method [6]. In particular, the normalization factors were re-scaled by the median of the normalized library sizes and then used to obtain the normalized read counts.

Finally, each cluster of DE genes was associated to a common specific GO term. To each of these GO terms, a random number of non-DE genes was also associated, ranging between 9 and 925. The remaining not-DE genes were randomly associated to other randomly chosen GO terms. R Packages *GO.db *and *org.Hs.eg.db *were used to define the DAG structure of GO terms and the GO annotations, respectively.

### Performance evaluation

*FunPat *was tested to evaluate its ability to: 1) recover false negatives in the selection of DE genes without decreasing the false discovery rate; 2) correctly cluster the genes associated to the same temporal pattern; 3) give reproducible results on independent replicates. The statistical significance of all the comparisons done was evaluated using two-sided Wilcoxon signed-rank test.

#### Selection of DE genes

Selection performance was assessed in terms of precision (number of true positives divided by the number of selected features) and recall (number of true positives divided by the number of true DE genes) in detecting the 120 simulated DE genes. *FunPat *selection performance was compared to edgeR and two existing methods specifically designed for time series expression data: maSigPro, using the new generalize linear model for the RNA-seq data [17], and the FPCA-based approach proposed in Wu and Wu [15]. In the comparison, we also considered the stand-alone application of the Bounded-Area method in order to evaluate if the integration of gene selection with the clustering step and the functional annotation is able to improve the recall without loss in precision.

EdgeR was applied to the data using the GLM application, by defining two factors for the model: one indicating the treatment/control samples, and the other indicating the corresponding time point, as suggested in [22].

MaSigPro applies two generalized linear regression steps to model gene expression in time series expression data. In particular, the former generates for each gene an ANOVA table and the related p-values; the latter is a stepwise regression analysis applied only to the genes with significant p-value. The goodness of fit of the obtained models, namely R^2^, can optionally be used to perform an additional gene selection step. In the evaluation of maSigPro on our simulated data we used the latest version adapted for RNA-seq data [17], considering the results obtained by both the first regression step (no threshold on R^2^) and setting a threshold on R^2 ^equal to 0.7 (maSigPro default setting). In both regression steps the same two factors defined for edgeR were considered for the generalized linear model.

Differently from the above methods, the FPCA-based approach [15] integrates principal component analysis into an hypothesis testing framework, identifying data-driven eigenfunctions representing the expression trajectories. The related test, publicly available at the Immune Modeling Community Web Portal repository [23] was used to perform the gene selection on our data.

#### Identification of temporal patterns

The ability to correctly associate the expression profiles to the corresponding simulated patterns was assessed in terms of clustering precision (C-precision) and recall (C-recall), defined as described in Figure 3. The two scores were calculated by matching each identified profile to one of the simulated patterns looking at the maximum intersection between the groups of genes identified by the clustering method and those assigned to a cluster by the simulation, respectively. C-precision was calculated as true positives, i.e. the number of genes in the intersection, divided by the number of genes associated to the identified profile; the C-recall was calculated as true positives divided by the number of genes (i.e. 20) associated to the simulated pattern. Figure 3 provides a toy example.

In addition to C-precision and C-recall, we also considered the normalized mutual information (NMI) to quantify the shared information between the simulated partition and the clustering results. Specifically, mutual information was calculated using the contingency table obtained by the true partition and the clustering results; since the mutual information has no upper bounds, its normalized version, ranging between 0 and 1, was used [24].

*FunPat *clustering performance was compared to the hierarchical and k-means clustering approaches and to a model-based method recently adapted for RNA-seq data [12]. The number of clusters obtained as output by *FunPat *was used to set the number of clusters for all the other methods.

#### Reproducibility of DE gene lists

As a further evaluation, we considered the reproducibility of the results to assess the ability of each method to detect the same DE genes when it is applied independently on available independent replicates. We applied *FunPat *and the other selection methods to each single replicate and evaluated the reproducibility of the resulting lists of DE genes in terms of intersection across the three replicates divided by the minimum list size, i.e. the smallest list size among the lists of DE genes identified for each replicate. The same evaluation was also applied to two different real datasets [16,18], focusing also on the ability of *FunPat *to obtain biologically interpretable results.

## Results

### Selection of DE genes

**Figure 4:**
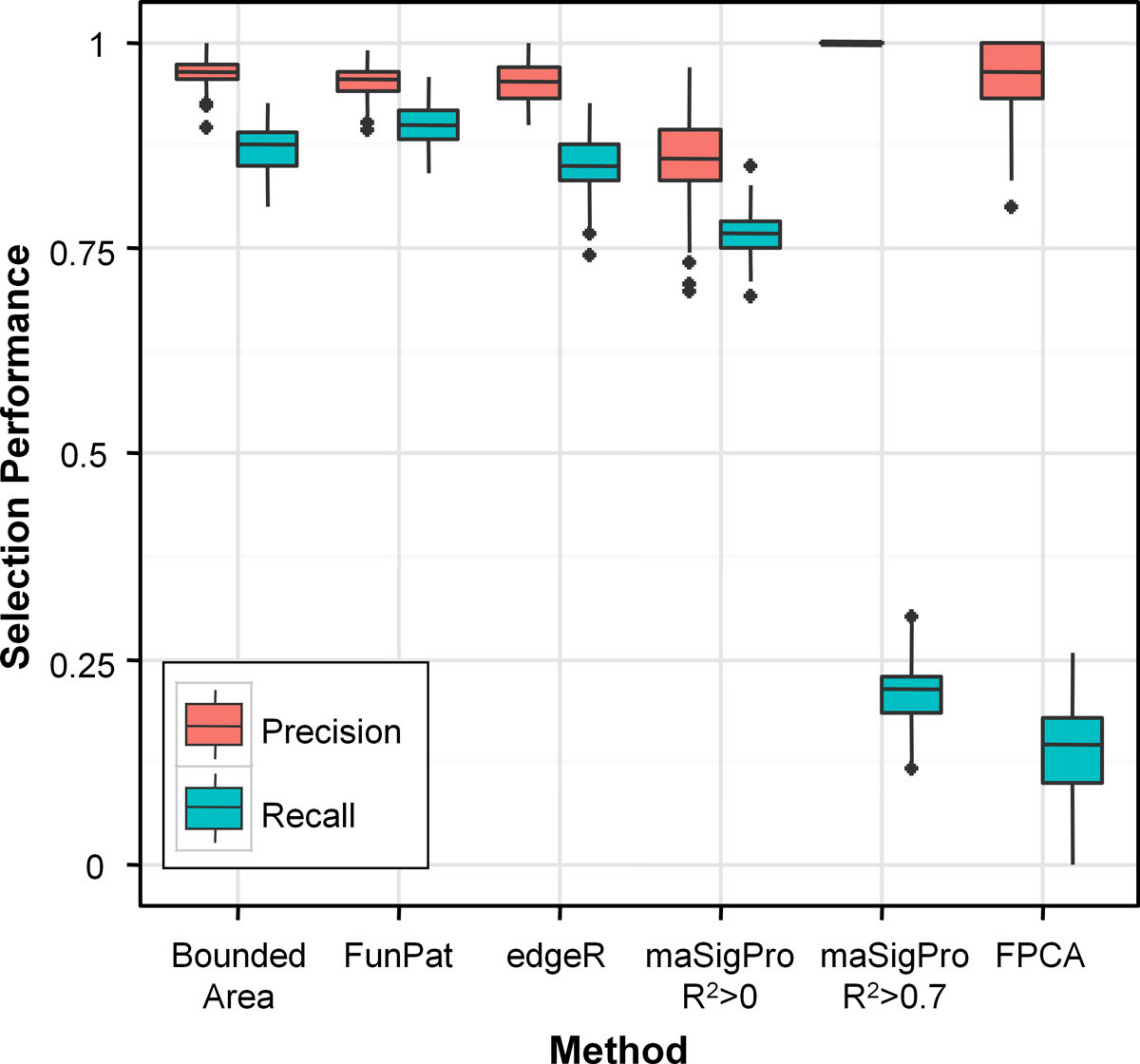
**Gene selection performance on simulated data**. Boxplots of precision and recall in selecting the 120 DE genes, comparing the list of DE genes provided by *FunPat *to those obtained from the Bounded-Area method, edgeR, maSigPro and FPCA.

The ability of *FunPat *and the other selection methods to select the simulated DE genes is displayed in Figure 4. *FunPat *is able to significantly increase the average recall with respect to the stand-alone application of the Bounded-Area method from 0.87 to 0.9 (p-value <1e-15), without changing the false discovery rate (average precision equal to 0.95). In terms of recall, *FunPat *outperforms also all the other selection methods considered. edgeR shows the same average precision of *FunPat *(p-value = 0.11), but the recall is significantly lower (0.85 on average, p-value <1e-15). Without any thresholds on R^2^, maSigPro obtains a precision equal to 0.86 and an average recall equal to 0.77, both significantly lower than those obtained by *FunPat *(p-value <1e-15 for both comparisons). When the default 0.7 threshold on R^2 ^is applied, the number of genes selected by maSigPro drastically drops (25 genes on average), obtaining an average precision equal to 1, but at the expense of the recall, equal to 0.21 on average. A similar result is obtained by FPCA, showing an average precision and recall equal to 0.96 and 0.14, respectively (17 genes selected on average).

### Identification of temporal patterns

**Figure 5:**
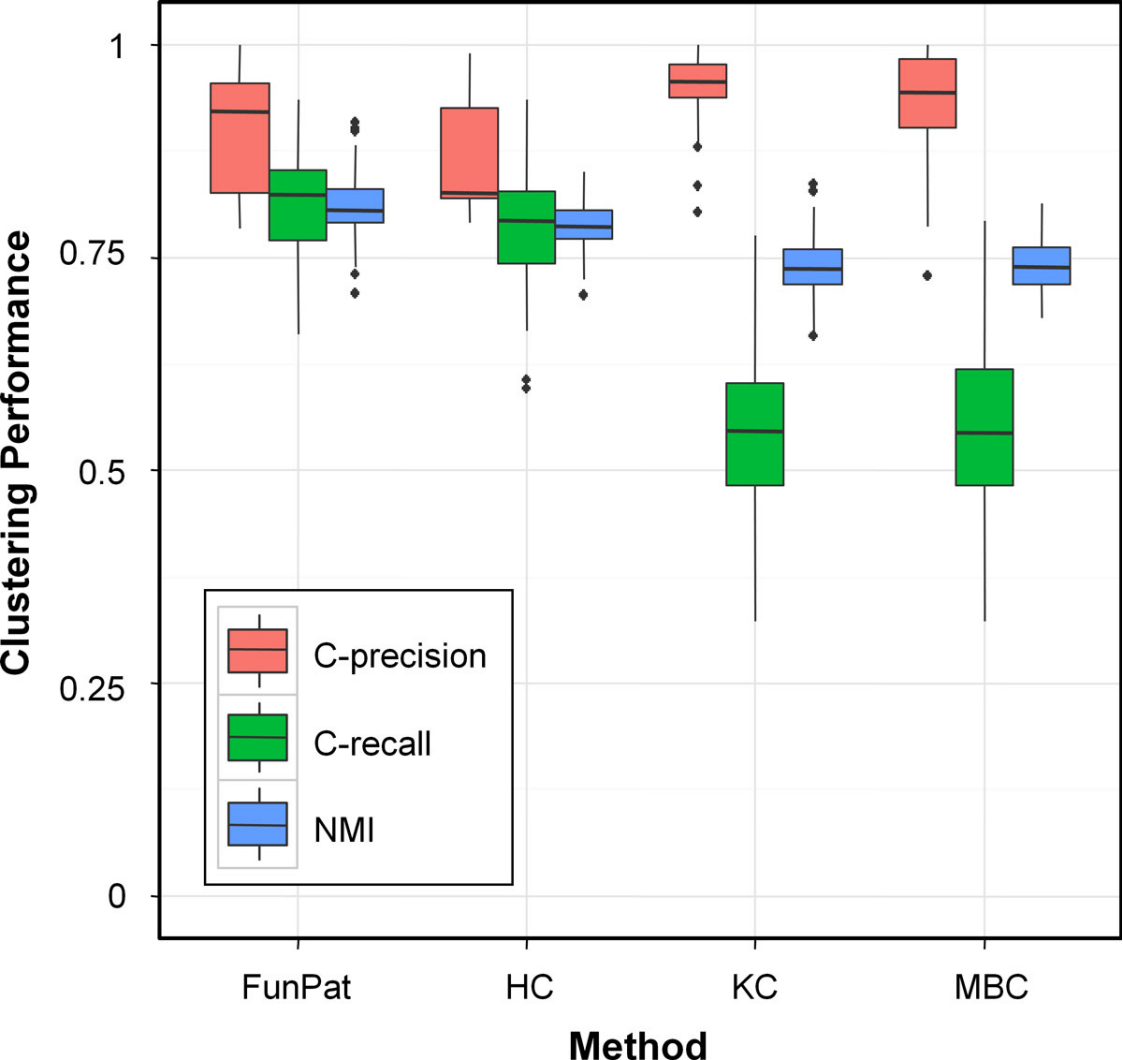
**Clustering performance on simulated data**. Boxplots of C-precision, C-recall and NMI in cluster identification, comparing *FunPat *to the hierarchical (HC), k-means (KC) and model-based (MBC) clustering.

The correct identification of the simulated patterns was tested against the hierarchical (HC), the k-means (KC), implemented in maSigPro, and the model-based clustering (MBC) method described in Si et al. [12]. In order to evaluate the clustering performance independently from the selection step, we assigned the genes selected by *FunPat *as input to the hierarchical, the k-means and the model-based clustering. The number of clusters obtained as output by *FunPat *was used to set the number of clusters for all the other methods. Boxplots in Figure 5 summarizes the obtained results. *FunPat *shows high C-precision (0.9) and C-recall (0.81) in cluster detection, both significantly higher with respect to HC (0.86, p-value = 1.83e-05, and 0.79, p-value = 3.41e-07, respectively). KC and MBC show significantly higher C-precision (0.95 and 0.93 respectively, p-value <2e-04), but at the expense of the C-recall, on average 0.54 and 0.55 respectively, compared to an average of 0.81 of *FunPat*, with p-values always lower than 1e-15. Moreover, *FunPat *shows the best NMI with average 0.81, compared to 0.79, 0.74 and 0.74 obtained by HC, KC and MBC, respectively (p-values always lower than 3e-07), reflecting that the DE genes selected by *FunPat *are well-distributed across the clusters.

### Reproducibility of DE gene lists

**Figure 6:**
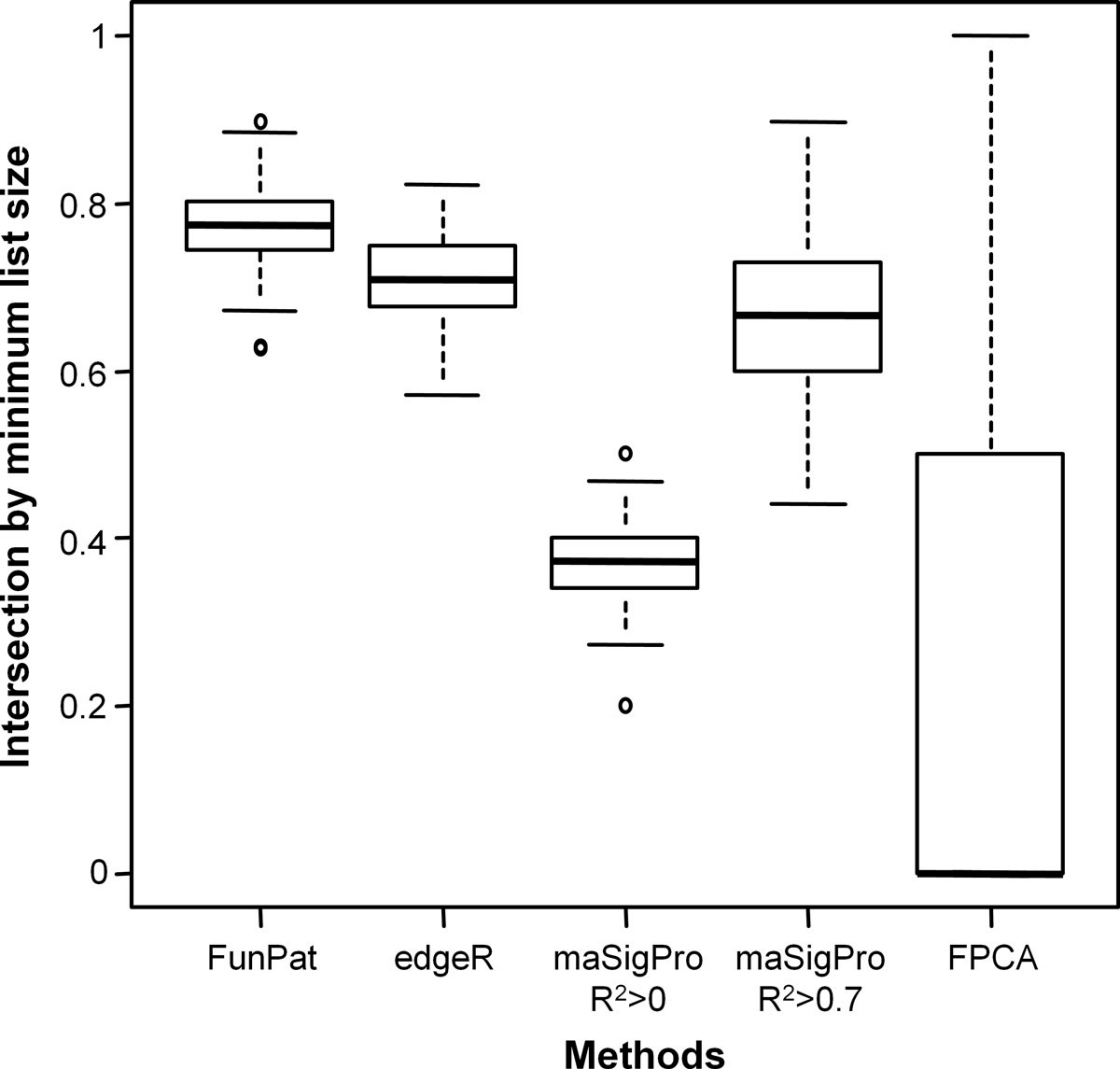
**Reproducibility of selected genes on simulated datasets**. Intersection divided by the minimum list size (i.e. the smallest list size among the lists of DE genes identified for each replicate), comparing, for each method, the three lists of DE genes obtained from the three replicates of each simulated dataset.

Considering the analysis applied to each single time series replicate, we wanted to assess, for each simulated dataset, whether the lists are also reproducible across the replicates. Figure 6 reports the boxplots of the intersection divided by the minimum list size for the 100 simulated datasets, comparing the three lists of DE genes obtained from the three replicates of each dataset. The best performing method is *FunPat *(0.77 on average). edgeR shows a lower reproducibility (0.71) as well as maSigPro (0.67 with the default settings, 0.38 without thresholds on R^2^), with p-values always below 4e-13 with respect to *FunPat*. Compared to all the other methods, the reproducibility of FPCA drastically drops, since in many datasets it does not select any genes.

### Application to real datasets

In order to better appreciate the various facets of the presented approach, *FunPat *and the other selection methods considered in the performance evaluation on the simulated data were also applied to two publicly available datasets. The first dataset represents the time varying B cell vaccine responses (days 0-10) analyzed by RNA-seq in five different subjects [16]. The study focuses on the identification of both common genes and patient-specific dynamics, since, on one hand, a number of immune response features should be common across subjects, on the other, it is known that influenza vaccines produce highly variable B cell responses among different individuals.

In the original study, each gene was tested for differential expression using the FPCA-based approach [15], using a 5% FDR threshold to account for multiple testing. The authors reported a union set of 6849 DE genes across the five subject, of which less than 1% belongs to the intersection set. Interestingly, three subjects that, differently from the other two, were vaccinated within the previous three years show much higher similarity with a union set of 5790 genes, of which around 13% (742 genes) are in common.

*FunPat *was applied to each subject independently, using a FDR threshold equal to 5% to define the seeds. GO annotations and GO DAG derived from R packages *org.Hs.eg.db *and *GO.db *respectively were considered. 4791 genes resulted differentially expressed across the five subjects. Of these, only 1.2% are in common among all the subjects, consistently with the original study (reporting 1% overlap). However, when considering the three previously vaccinated individuals, *FunPat *identified a union set of 4447 genes of which 21% (896 genes) are in common across the subjects, in comparison with the 13% obtained in the original study. Considering the list of 896 genes in common, about 60% (445 genes) were selected in the same three subjects also in [16].

Applied to the same dataset, maSigPro selected 9374 and 2205 genes without thresholds on R^2 ^and with the default threshold of 0.7, respectively. These results are characterized by a very low intersection between the gene lists, with no more than 0.2% in common among all the subjects, and no more than 2% considering the three subjects previously vaccinated. The high overlap observed with both *FunPat *and FPCA among the three subjects is not achieved by maSigPro, which provides a signature of 190 and 1 genes in the intersection of the gene lists for R^2^>0 and R^2^>0.7, respectively. In the former list, only 22 genes are in common with the signature reported in [16]. The selected gene in the latter list, CDCA2, results selected also in [16].

**Figure 7:**
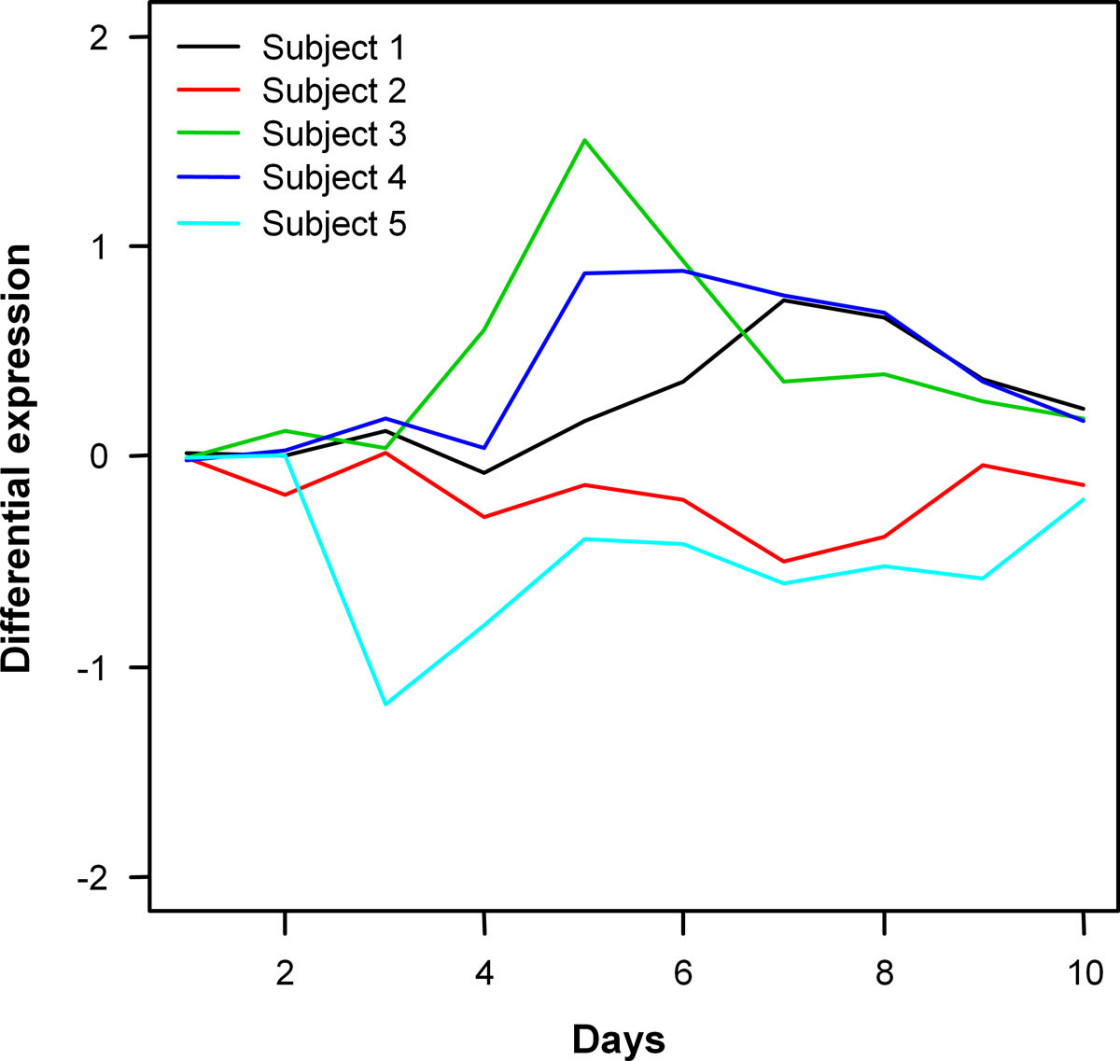
**Cell differentiation patterns**. Patterns identified by *FunPat *across the five subjects for the GO biological process *Cell differentiation*.

Differently from the simulated data, for which we have a defined list of true DE genes as a benchmark to compare the methods, here we do not know the real truth. However, in the original study the signature of 742 genes in common among the three previously vaccinated subjects were shown in strong correlation with migrating plasma cells. In a memory response to influenza vaccination, resting memory B cells are induced to differentiate through several stages into plasmablast and long-lived plasma cells, thus a higher overlap among the three subjects previously vaccinated is expected. Compared to maSigPro, *FunPat *is able to describe a better correspondence among these subject, in accordance with the validation made in the original study. As a further support to this result, *FunPat *is also able to indentify subject-specific patterns in common Gene Sets across the five subjects. As an example, the biological process *Cell differentiation *and the associated dynamic patterns are shown in Figure 7. Cell differentiation is an important process in vaccination, since B cells are induced to differentiate through several stages into long-lived plasma cells. Subjects 1, 3 and 4, vaccinated within the previous three years, share a similar dynamic response with respect to the other two subjects in this Gene Set. Interestingly, these patterns are characterized by different timing of peaks of expression level and by less or more gradual changes in gene expression, showing subject-specific temporal responses highlighted also in the original study. Compared to FPCA and maSigPro, which do not use prior information, *FunPat *is able to automatically associate these patterns to their common functional annotation, thus allowing an easier interpretation of the results.

Unfortunately, it was not possible to test edgeR in this dataset since un-normalized count data, required by this method, are not provided.

As a further assessment of the reproducibility of the gene lists, we considered a second real dataset representing the gene expression changes at defined stages during pancreatic endocrine differentiation of human embryonic stem cells [18]. The authors kindly provided us the count data in order to use also edgeR. Experiments were performed on two independent biological replicates, monitoring the temporal differentiation pattern from human embryonic stem cells (hESCs) towards the pancreatic fate. Here, we focused on the first five developmental stages, comparing the temporal expression profiles with respect to a reference ground state, set to the hESCs population. We applied *FunPat*, edgeR, maSigPro and FPCA to both biological replicates independently, normalizing the count data using the TMM approach. Since for the time series based on developmental stages the number of resulting DE genes and biological mechanisms involved is usually high, here we wanted to focus on the most important processes characterizing the temporal patterns observed. Therefore, we decided to use Bonferroni correction on p-values of edgeR, FPCA and those used to define the seed genes in *FunPat*. maSigPro was applied using the 0.7 threshold on R^2 ^in order to deal with the most differentially expressed genes. GO annotations and GO DAG derived from R packages *org.Hs.eg.db *and *GO.db *respectively were used in *FunPat*. As done for the simulated data, the overlap between the lists was calculated in terms of intersection divided by the minimum list size, i.e. the smallest list size among the lists of DE genes identified for each replicate.

Comparing for each method the two lists of DE genes resulted from the replicates, both *FunPat *and edgeR show the highest overlap (0.76 in both methods) with respect to maSigPro (0.52) and FPCA (0). Results confirm what observed in the simulated data and, considering also the results obtained in the first real dataset, it seems that, differently from maSigPro and FPCA, FunPat is able to provide more stable lists of DE genes, thus increasing the biological interpretability of the results.

## Discussion

A novel analysis framework, implemented in the R package *FunPat*, was developed to search for the main temporal patterns in classes of functional Gene Sets and to improve the gene selection by integrating the statistical evidence of differential expression with the information on the temporal profiles and the functional annotations. In particular, *FunPat *implements a differential expression analysis able to consider differences between two experimental conditions, taking into account the entire temporal expression profiles. The method is based on a model of the biological-plus-technical variability and of its dependence on the average gene expression; it is not constrained by any specific statistical distributions, thus allowing its application to RNA-seq data pre-processed in different ways and, in general, to different technologies. It is important to note that, although the method is robust to different data pre-processing approaches, data need to be normalized before using *FunPat *in order to correct for differences in sequencing depth and guarantee an accurate estimate of the biological-plus-technical variability after the removal of systematic biases.

In a conventional analysis, the user selects the genes using some correction method to adjust the p-values for multiple testing and then applies the clustering independently with respect to the selection step. A side effect of this approach is that the clustering is too constrained by the results obtained in the selection step, where the need to control the type I error rate in a multiple testing condition leads to very small significant thresholds, thus increasing the number of false negatives. To overcome this drawback, *FunPat *combines the information on p-values with both functional annotations and characteristic temporal pattern associations, thus decreasing the number of false negatives without significantly increasing the false discovery rate. The clustering method is based on a linear model, does not require the user to fix the number of clusters and is not computationally demanding. Since the model is purely based on a least squares method, the algorithm is flexible for applications to data from different platforms and/or processed in different ways.

Finally, significant genes are associated to the most informative Gene Sets, avoiding the redundancy of information on Gene Sets representing general biological functions. In particular, *FunPat *exploits, when available, the hierarchical structure of the annotations starting the search of the temporal patterns from the most specific functional terms and removing the selected genes from the ancestors, as originally proposed by Alexa et al. [21] in the context of functional enrichment. However, it is worth noting that *FunPat *does not perform any enrichment analysis on the selected genes, but only exploits annotations to select DE genes characterized by both a common temporal pattern and a common biological function.

Considering the application to the simulated data, both selection and clustering performance confirm that *FunPat *is able to provide, with respect to all the other methods considered in this study, the best trade-off between precision *vs*. recall and C-precision *vs*. C-recall, respectively. Moreover, *FunPat *shows the best reproducibility of the identified lists of DE genes with respect to the other methods. More specifically, *FunPat *shows almost comparable precision with respect to edgeR, but it outperforms this latter in terms of recall. *FunPat *also outperforms maSigPro in terms of both precision and recall when no thresholds on R^2 ^are imposed. On the other hand, the choice of the default setting (threshold on R^2 ^equal to 0.7) leads to a precision equal to 1 at the expense of a marked drop in recall, consistently below or equal to 0.3 for all the simulated datasets.

Compared to *FunPat*, edgeR and maSigPro, FPCA shows the worst performance in terms of recall and reproducibility, selecting few DE genes, although with good precision. Even if both FPCA and maSigPro with R^2^>0.7 show a higher precision with respect to *FunPat *and edgeR (Figure 4), the reproducibility assessment shows that these methods tend to select different lists of true positive genes across different biological replicates of the same dataset.

As regards the identification of the temporal patterns, *FunPat *outperforms in terms of C-recall with respect to all the other methods. Even if k-means and the model-based method show a higher C-precision, they present the lowest average recall, thus not providing the same trade-off between C-precision and C-recall of *FunPat*. As a further support of this result, *FunPat *outperforms all the methods also in terms of NMI score, thus highlighting the ability of *FunPat *to provide more well-distributed clusters with respect to k-means and the model-based method, characterized by the lowest average NMI scores.

Finally, focusing on the definition of seeds and candidates defined by *FunPat *using the p-values obtained by the Bounded-Area method, one may wonder if the selection performance would be affected by a selection method different from the Bounded-Area. Considering the simulated data, when three replicates are available, the performance obtained using either edgeR or the Bounded-Area method are almost comparable (average precision 0.95 and 0.96, average recall 0.85 and 0.87, respectively). When the two methods are applied to a single replicate, the Bounded-Area method, which was specifically designed for time series in data-poor conditions, is able to select a higher number of genes (77 on average) than edgeR (64), maintaining, as edgeR, good precision, not statistically different with respect to a required false discovery rate equal to 5%, but showing a higher recall (0.6 with respect to 0.5 obtained with edgeR). Our conclusion is that different methods can be used to assign the input p-values to the list of analyzed transcripts and to define seeds and candidates, but it is advisable to base the choice on the dataset characteristics. In particular, it is worth noting that *FunPat *outperforms the other methods also when data suffers from missing replicates. Obtained results, illustrated in Additional File 3, show a pattern similar to that observed in Figure 4 and emphasize the higher gain in recall of *FunPat *with respect to all the other methods. This result is also supported by the application of *FunPat *to the two real datasets considered in this study, for which the methods were always applied to single replicates.

## Conclusions

*FunPat *is an R package that integrates gene selection, clustering and functional annotations into a single analysis framework, providing clusters of DE genes associated to temporal patterns and specific biological terms. Tested on simulated time series data, *FunPat *shows better performance with respect to both the selection and the clustering step. The integration of the selection and the clustering step is able to improve the recall without altering the false discovery rate with respect to a stand-alone selection step. Moreover, the ability to identify different time series expression patterns is higher than that observed using hierarchical, k-means or model-based clustering approaches specifically designed for RNA sequencing data. Finally, when data are characterized by missing experimental replicates, *FunPat *is able to provide highly reproducible lists of DE genes. The application to two real datasets confirms the ability of *FunPat *to select differentially expressed genes with high reproducibility on different time series expression data, thus indirectly confirming the ability of *FunPat *to select genes with high precision and recall.

## Competing interests

The authors declare that they have no competing interests.

## Authors' contributions

TS helped in conceiving the study, designed *FunPat *pipeline code and prepared the R package, did the analyses on simulated and real data and wrote the manuscript. FF designed the simulation code and generated the normalized simulated datasets. BDC conceived and supervised the study, designed *FunPat *pipeline code and wrote the manuscript. All authors have read and approved the manuscript in its current form.

## Supplementary Material

Additional file 1 **FunPat linear model-based clustering algorithm**. Detailed description of the clustering method used in *FunPat *pipeline for searching the temporal patterns, including the pseudo-code of the algorithm. Click here for file

Additional file 2 **Output examples of HTML reports**. HTML reports generated by *FunPat *displaying the output of the Bounded-Area method and the temporal pattern profiles associated to each Gene Set. Click here for file

Additional file 3 **Results of selection and clustering performance on single replicate**. Description of the selection and clustering performance of the methods when they are applied independently on the available replicates. Click here for file

## Declarations

Publication of this article has been funded by PRAT 2010 CPDA101217 ("Models of RNA sequencing data variability for quantitative transcriptomics").

This article has been published as part of *BMC Genomics *Volume 16 Supplement 6, 2015: Proceedings of the Italian Society of Bioinformatics (BITS): Annual Meeting 2014: Genomics. The full contents of the supplement are available online at http://www.biomedcentral.com/bmcgenomics/supplements/16/S6.
