# Supplementary material for: FunPat: function-based pattern analysis on RNA-seq time series data
Source: BMC Genomics. 2015 Jun 1;16(Suppl 6):S2. doi: 10.1186/1471-2164-16-S6-S2 (PMC4460925; doi:10.1186/1471-2164-16-S6-S2)
Supplement: Additional file 1 — FunPat linear model-based clustering algorithm. Detailed description of the clustering method used in FunPat pipeline for searching the temporal patterns, including the pseudo-code of the algorithm. [file 1471-2164-16-S6-S2-S1.docx]

Additional File 1

Model-based clustering algorithm

The pattern analysis is performed using a linear model-based clustering, which searches for a cluster of genes whose time series expression profile $X=<x\left( 1 \right),\ldots,x\left( M \right)>$ can be modeled by the following equation:

$$X=k\cdot P+q+\sum$$

where $P=<p\left( 1 \right),\ldots,p\left( M \right)>$ is the characteristic temporal pattern, *k* and *q* are the gene-specific parameters of the model and Σ is the covariance matrix of the error. The algorithm iteratively performs a gene-specific parameter identification step (M step) and a temporal pattern search (E step), using an Expectation-Maximization approach. A pseudocode of the algorithm is illustrated in Figure 1. At the first iteration, the temporal pattern *P* is initialized with the time series expression profile of a seed gene given as input. Using the results from the Gene Ranking module, the seed with the lowest p-value is considered to initialize *P*. Given the gene expression data *X* and the pattern *P*, in the M step the parameters *k* and *q* are identified for each gene using weighted least squares method, defining the cluster *C* of genes fitting the pattern P. The membership of a gene in a cluster *C* is based on: 1) a goodness of fit test to P by applying a runs test and a chi-square test to the residuals, 2) a statistical assessment in comparison to a at profile. The user can fix a significance level α for the tests and only genes with significant p-values are kept in the cluster. In the E step, *P* is identified at each sampling time, applying again the weighted least squares, using the parameters *k* and *q* of the genes belonging to *C* and estimated at the M step. All the analyzed genes go again through the M step, so to update the estimation of the parameters *k* and *q* and re-define the cluster membership based on the newly estimated pattern *P*. The algorithm re-iterates all the steps until the genes belonging to *C* do not change or a maximum number of iterations is reached. Finally, the mean pattern *P_m_* representing the average time series expression profile across all genes joining the cluster *C* is defined using the parameters *k_m_* and *q_m_* estimated at the last iteration:

$$P_{m}=k_{m}\cdot P+q_{m}$$

with

$$k_{m}=\frac{k}{\sum_{i=1}^{r} k_{i}}$$

$$q_{m}=-\frac{\sum_{i=1}^{r} q_{i}}{\sum_{i=1}^{r} k_{i}}\cdot k+q$$

where $\sum_{i=1}^{r} k_{i}$ and $\sum_{i=1}^{r} q_{i}$ indicating the mean *k* and *q* values across the *r* genes belonging to *C*. After identifying a pattern and a cluster of genes, these are removed and the entire procedure is repeated again on the remaining genes, until the number of genes is no sufficient to create a new cluster or no other significant patterns can be discovered.

**
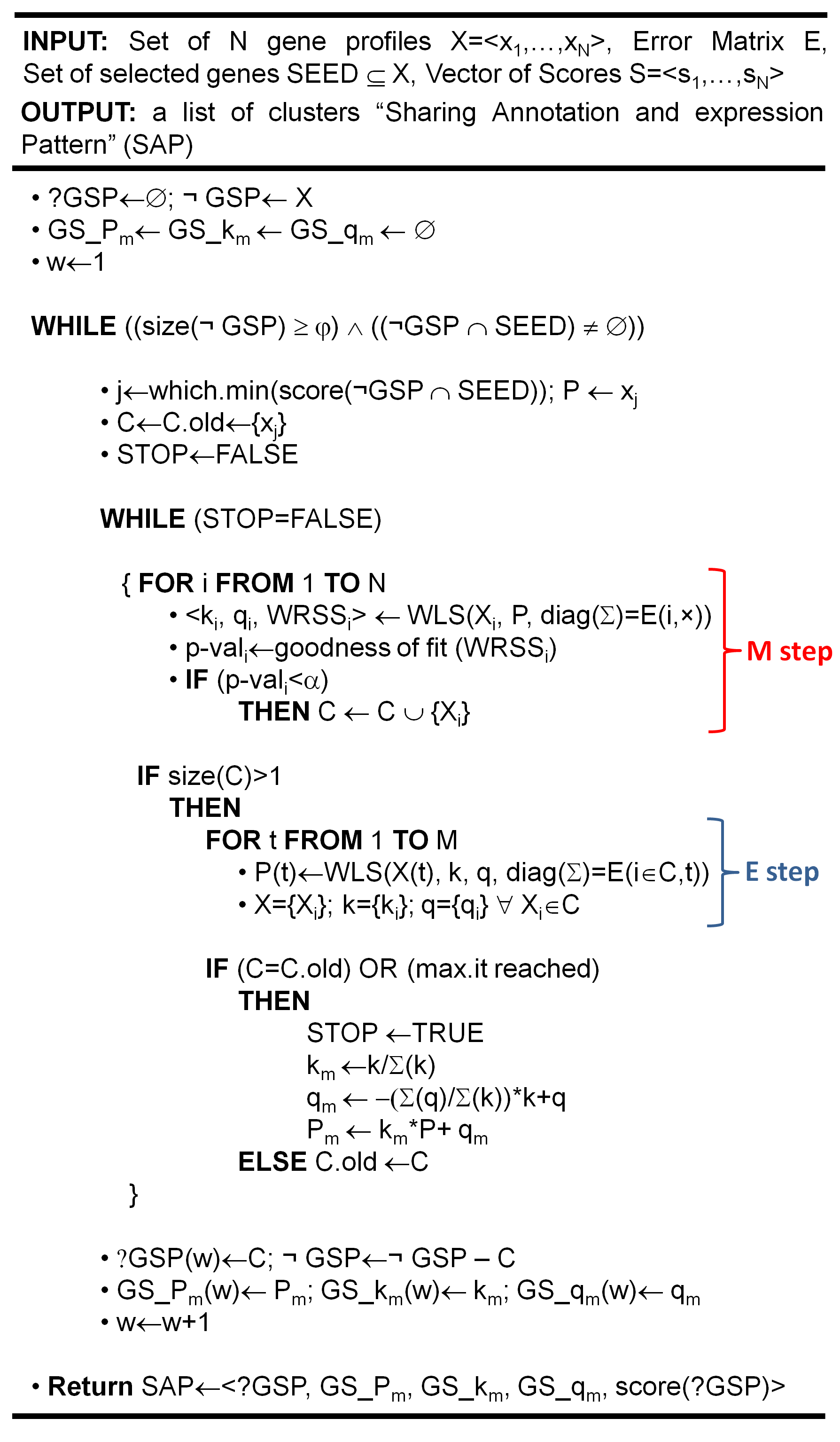
**

**Fig 1. Pseudo-code description of the model-based clustering approach.** The algorithm iteratively searches the main characteristic patterns starting from a set of N gene profiles and a set of seed genes provided as input. The algorithm indentifies a list of clusters ?GSP, each characterized by a pattern *P_m_* by updating the set of genes which do not fit any pattern (¬GSP). If the size of ¬GSP is greater than a minimum cluster size fixed by the user AND there is at least a seed gene available to initialize a pattern *P*, the algorithm searches for new patterns. The M step identifies the fitting parameters *k* and *q* and, if there are at least two genes with a p-value lower then a fixed significance threshold α, the E step is performed updating the pattern *P*. The two steps are re-iterated until the set of genes does not change or a maximum number of iterations (*max.it*) is reached, defining the cluster *C*, the mean pattern *P_m_* and the parameters *k_m_* and *q_m_*. ¬GSP is updated and the entire procedure is then applied again to ¬GSP, until it does not contain a sufficient number of genes or no other significant patterns can be discovered. The algorithm returns the set of genes belonging to each cluster ?GSP, the set of identified patterns *GS_P_m_* and the estimated model parameters *GS_k_m_* and *GS_ q_m_*. If available, the related ranking scores (i.e. the p-values resulting from the Gene Ranking module) used for the initialization of the patterns are also returned.
