# Supplementary material for: FunPat: function-based pattern analysis on RNA-seq time series data
Source: BMC Genomics. 2015 Jun 1;16(Suppl 6):S2. doi: 10.1186/1471-2164-16-S6-S2 (PMC4460925; doi:10.1186/1471-2164-16-S6-S2)
Supplement: Additional file 2 — Output examples of HTML reports. HTML reports generated by FunPat displaying the output of the Bounded-Area method and the temporal pattern profiles associated to each Gene Set. [file 1471-2164-16-S6-S2-S2.doc]

Additional File 2

Output examples


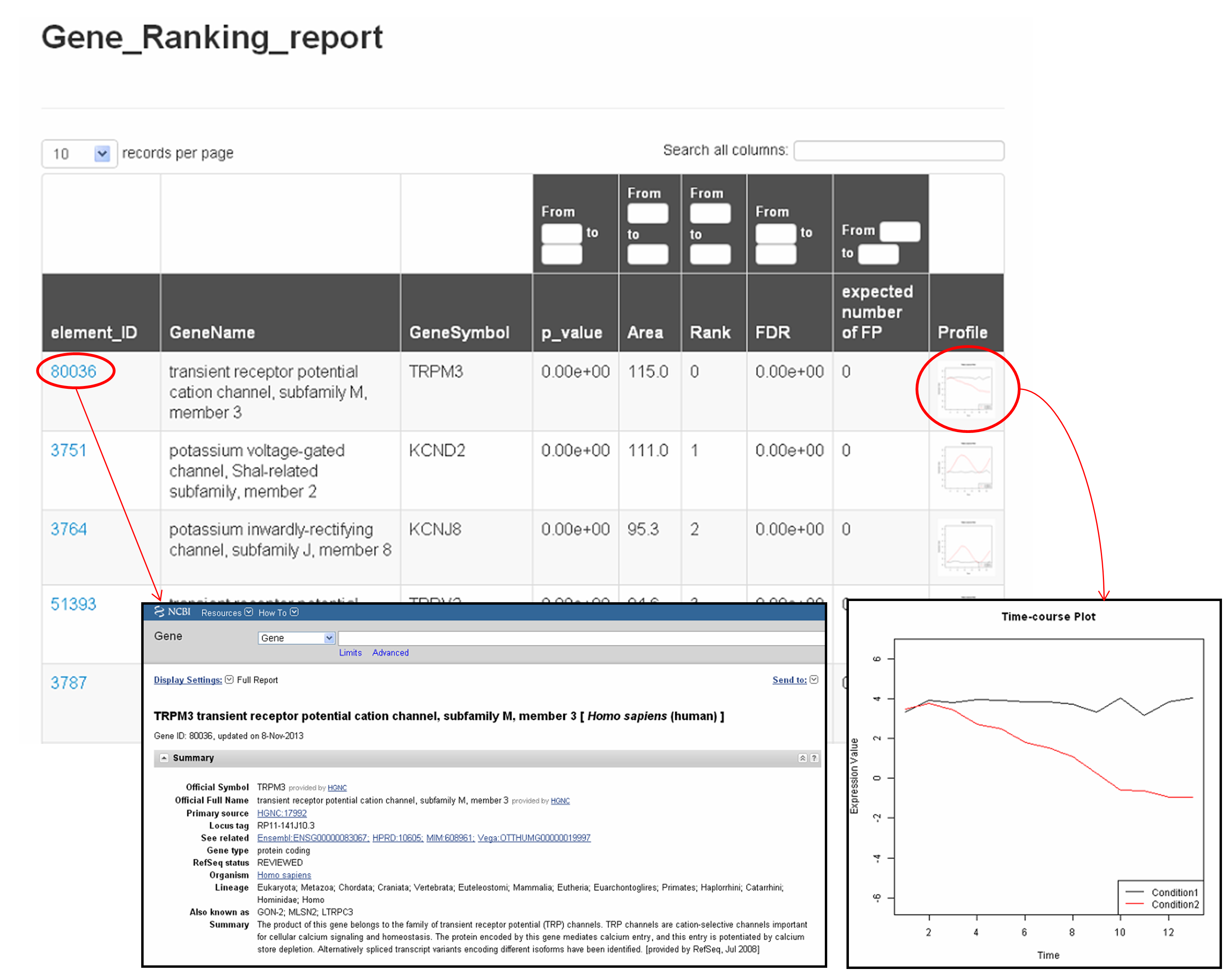


**Fig 1. Example of HTML page resulting for the Gene Ranking module.** If available, the Entrez identifiers are linked to the corresponding NCBI web page and the plot of the related time series profiles in the two experimental conditions is displayed.


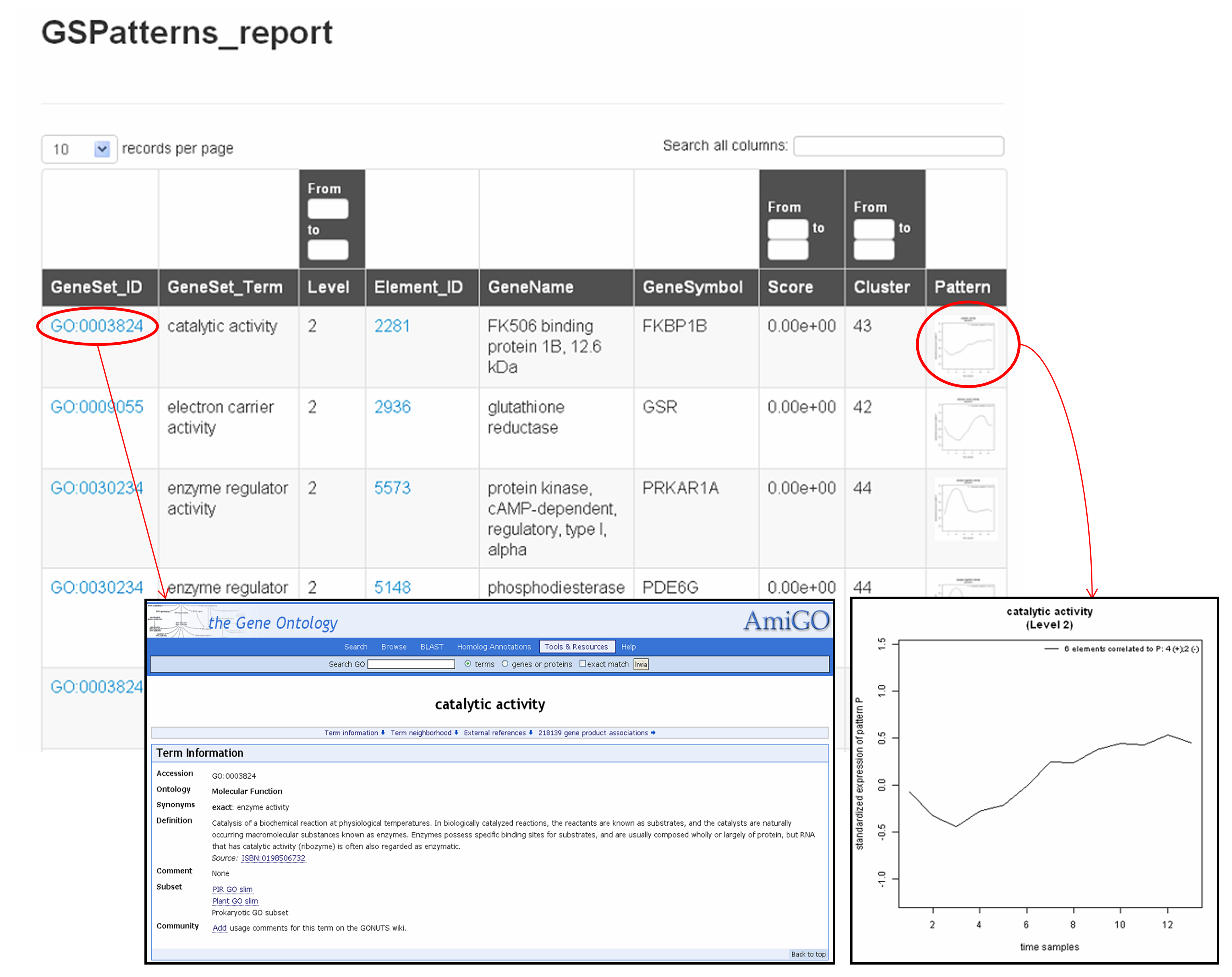


**Fig 2. Resulting HTML page reporting the Gene Set-specific patterns identified in the Temporal Pattern Analysis module.** If available, the Gene Set identifiers matching with Gene Ontology terms are linked to the corresponding AmiGO web page and the plot of the related temporal patterns is displayed.
