# Supplementary material for: FunPat: function-based pattern analysis on RNA-seq time series data
Source: BMC Genomics. 2015 Jun 1;16(Suppl 6):S2. doi: 10.1186/1471-2164-16-S6-S2 (PMC4460925; doi:10.1186/1471-2164-16-S6-S2)
Supplement: Additional file 3 — Results of selection and clustering performance on single replicate. Description of the selection and clustering performance of the methods when they are applied independently on the available replicates. [file 1471-2164-16-S6-S2-S3.doc]

Additional File 3

## Selection of DE genes at single time series replicate

The ability of *FunPat* and the other selection methods to select the simulated DE genes when applied independently to each single time series replicate is displayed in Figure 1. *FunPat* shows the highest recall, 0.7 on average, with respect to the other methods: 0.6 for the Bounded-Area method, 0.52 for maSigPro with no threshold on R^2^, 0.5 for edgeR and 0.25 for maSigPro with default setting. FPCA is not able to select at least one gene in all the datasets analyzed, selecting at most 11 genes per dataset. *FunPat* preserves a high precision (0.92 on average), slightly lower than the nominal false discovery rate as also edgeR, characterized by a higher precision with respect to *FunPat* (0.94) but significantly lower than the false discovery rate. However, the relative gain of *FunPat* in terms of recall is strongly higher, with a p-value always below 1e-15 in all the comparison with the other methods.


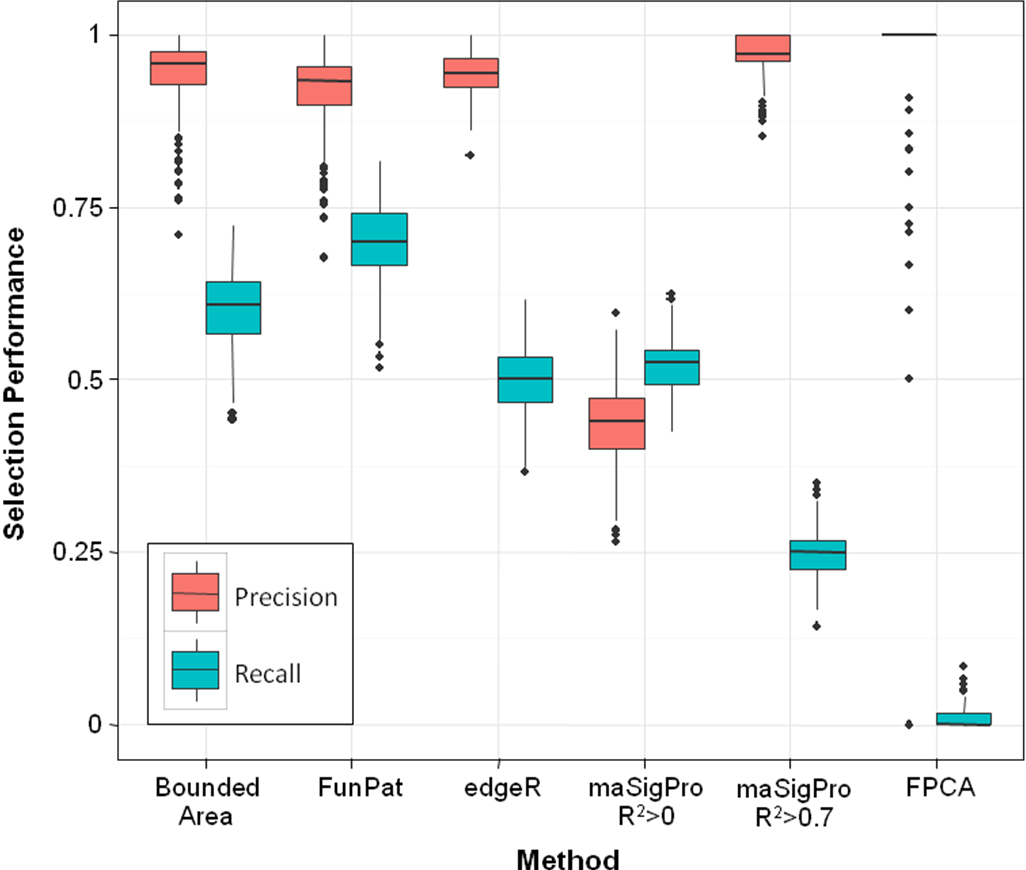


**Fig 1. Selection performance at single time series replicate.** Boxplots of precision and recall in selecting the 120 DE genes, comparing the list of significant genes provided by *FunPat* to those obtained from the Bounded-Area method, edgeR, maSigPro and FPCA.

## Identification of temporal patterns at single time series replicate

In the application to each single time series replicate, FunPat shows clustering performance similar to what observed using the three replicates. In particular, the differences of performance in terms of C-recall are more emphasized: FunPat shows the best average C-recall (0.68) with respect to HC (0.5), KC (0.37) and MBC (0.4). On the other hand, FunPat preserves a good C-precision (0.86), still significantly higher than HC (average C-precision 0.84, p-value<0.02). KC and MBC show the best C-precisions (on average 0.88 and 0.91 respectively), but these results are modulated by very low C-recalls. Finally, FunPat shows the best performance also for the NMI score (0.75 on average) with respect to HC (0.68), KC (0.64) and MBC (0.7), with p-value<1e-15 for all the comparisons. This latter result supports and summarizes the main differences observed in the previous performance, highlighting that FunPat is able to provide the best trade-off between C-precision and C-recall by assigning the genes belonging to different simulated patterns to different clusters.


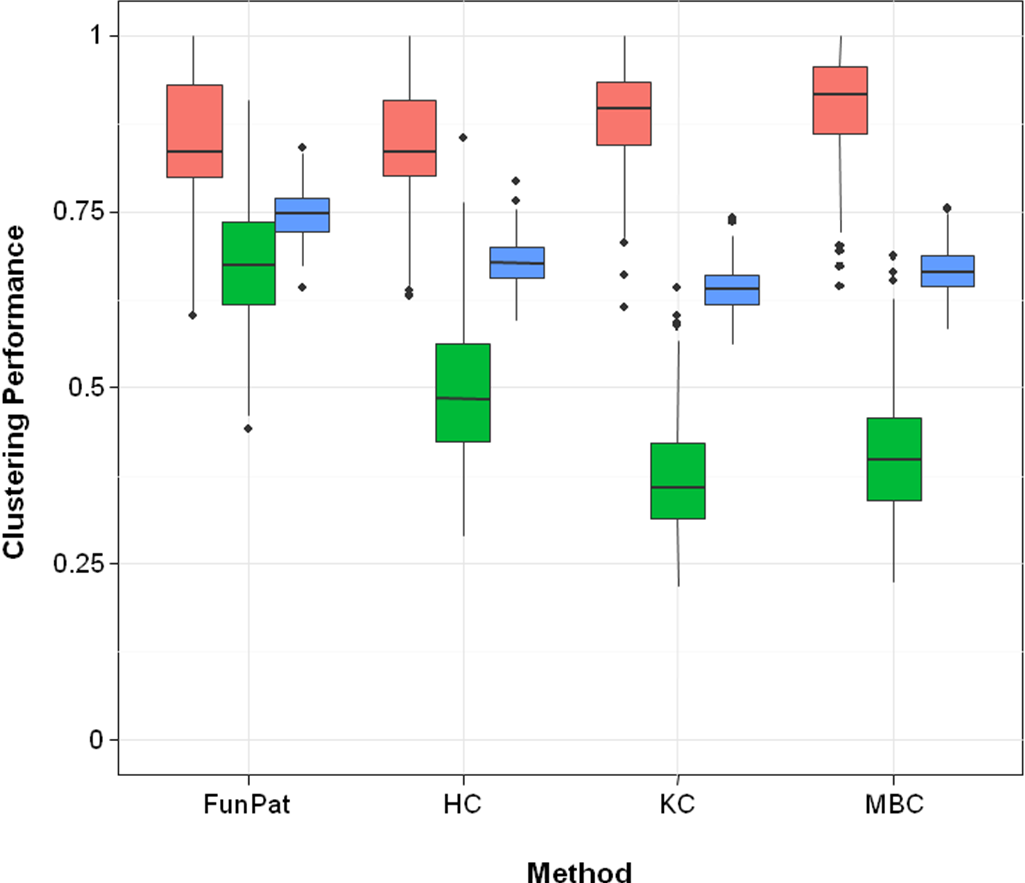


**Fig 2. Clustering performance at single time series replicate.** Boxplots of C-precision, C-recall and NMI in cluster identification, comparing *FunPat* to the hierarchical (HC), k-means (KC) and model-based (MBC) clustering.
